# Transcriptome Analysis of Pepper Leaves in Response to Tomato Brown Rugose Fruit Virus Infection

**DOI:** 10.3390/plants14091280

**Published:** 2025-04-23

**Authors:** Boshen Zhang, Donghai Wang, Mangle Chen, Jiali Yang, Junmin Li, Jianping Chen, Fei Yan, Shaofei Rao

**Affiliations:** 1 College of Agriculture and Biotechnology, Zhejiang University, 866 Yu Hang Tang Road, Hangzhou 310058, China; 2 State Key Laboratory for Quality and Safety of Agro-Products, Key Laboratory of Biotechnology in Plant Protection of MARA, Zhejiang Key Laboratory of Green Plant Protection, Institute of Plant Virology, Ningbo University, Ningbo 315211, China

**Keywords:** tomato brown rugose fruit virus, chili pepper, transcriptome analysis

## Abstract

Chili pepper (*Capsicum annuum* L.) is a very important vegetable crop, commonly used as a spice or seasoning in various dishes. With the growth of the global population, the demand for chili peppers has also increased exponentially. Tomato brown rugose fruit virus (ToBRFV) is an emerging tobamovirus that has spread to dozens of countries worldwide. Its infection in chili peppers can severely impact yield and quality, posing a significant threat to the chili pepper industry. However, the transcriptional response of chili peppers to ToBRFV infection has not been studied yet. This research utilized RNA-Seq technology to analyze the transcriptional profiles of chili pepper leaves (‘Haonong 11’) 13 days post-infection with ToBRFV or mock treatment, identifying a total of 1468 differentially expressed genes (DEGs), of which 1366 were upregulated and 102 were downregulated. Gene Ontology (GO) and Kyoto Encyclopedia of Genes and Genomes (KEGG) enrichment analyses indicated that the DEGs were involved in biological processes such as defense response, response to reactive oxygen species, protein folding, and plant-pathogen interaction. Twelve DEGs were validated by RT-qPCR, with their expression trends consistent with the transcriptome data, indicating the reliability of the high-throughput data. Our systematic analysis provides a molecular basis for the response of chili pepper leaves to ToBRFV infection at the transcriptomic level and offers potential candidate genes for further research into the interaction mechanisms between ToBRFV and plant hosts.

## 1. Introduction

Chili pepper (*Capsicum annuum* L.) is an annual or perennial plant belonging to the Solanaceae family, native to the tropical regions of Central and South America [1]. It is an important vegetable crop cultivated primarily for several purposes: consumption as a fresh vegetable, use as a spice after drying, extraction of edible pigments and flavorings, and derivation of other compounds for medicinal or industrial applications [2]. According to data released by the Food and Agriculture Organization (FAO) of the United Nations, global chili pepper production has gradually increased from 2018 to 2022. In 2022, Asia had the highest chili pepper production among all continents (followed by Africa, the Americas, Europe, and Oceania), contributing to over 70% of the world’s chili pepper production during this five-year period [3].

Pests and diseases severely limit chili pepper cultivation worldwide, causing significant damage to both the quality and quantity of production. Common diseases include Phytophthora blight and anthracnose, while harmful insects such as whiteflies, tobacco budworms, and thrips are also prevalent [2,4]. Additionally, it is known that 45 plant viruses can infect chili peppers, posing a threat to chili crops [5]. Moury and Verdin reviewed the main viruses affecting chili peppers in the Mediterranean basin, which include tobamovirus, potato virus Y (PVY), tobacco etch virus (TEV), cucumber mosaic virus (CMV), and tospoviruses [6]. Li et al. conducted a multi-year, multi-site survey of viruses infecting chili peppers in Yunnan Province, China, and found the detection rates from highest to lowest as follows: tomato spotted wilt virus (TSWV), pepper vein yellows virus (PeVYV), CMV, chili veinal mottle virus (ChiVMV), tomato mosaic virus (ToMV), tobacco vein distorting virus (TVDV), pepper mild mottle virus (PMMoV), tomato mottle mosaic virus (ToMMV), tobacco mosaic virus (TMV), tobacco mild green mosaic virus (TMGMV), broad bean wilt virus 2 (BBWV2), tobacco bushy top virus (TBTV), and wild tomato mosaic virus (WTMV) [7].

Tomato brown rugose fruit virus (ToBRFV) is a recently identified tobamovirus, first discovered in the spring of 2015 in greenhouse-grown tomatoes in Jordan, with its initial outbreak traced back to 2014 in Israel [8,9]. Tomato and pepper are the two natural host crops for ToBRFV. The virus has since spread to numerous countries across the Americas, Asia, Africa, and Europe, causing significant economic losses to local agricultural production [10,11]. It is currently regarded as a globally significant quarantine pest.

ToBRFV is a single-stranded, positive-sense RNA virus (+ssRNA) with approximately 6400 nucleotides, encoding four proteins. Among these, two are replication-associated proteins: p126 and p183. The other two proteins are the ~30 kDa movement protein (MP) and the ~17.5 kDa coat protein (CP). P126 and p183 are directly translated from the genomic RNA (with p183 produced through a stop codon read-through strategy), while MP and CP are translated from subgenomic RNAs. MP is essential for the cell-to-cell movement of the virus, and CP is involved in viral particle assembly and long-distance movement [8,9,10,11]. Like most tobamoviruses, ToBRFV is primarily transmitted through mechanical contact and can also spread over long distances via external seed contamination [10,11,12,13].

When pepper plants are infected by ToBRFV, it results in leaf wrinkling and yellow mottling, stunted growth and development of seedlings, and the appearance of small yellow to brown wrinkled spots and necrotic lesions on the fruits [11]. The *L* allele located on chromosome P11 of pepper encodes a coiled-coil, nucleotide-binding, leucine-rich repeat (CC-NB-LRR) resistance protein, which mediates resistance to tobamoviruses such as TMV, ToMV, TMGMV, and PMMoV. The CP encoded by tobamoviruses acts as an avirulence factor [14,15]. Studies have found that ToBRFV can infect pepper varieties carrying the *L1* or *L2* alleles, indicating that ToBRFV has overcome the resistance mediated by *L1* or *L2*. In pepper plants carrying the *L3* and *L4* alleles, inoculation with ToBRFV triggers a hypersensitive response (HR), a typical resistance reaction. However, this resistance mechanism is inactivated at high temperatures (32 °C and above) [16].

Viral infection of cells leads to rapid and extensive reprogramming of gene expression patterns in the host cells. Due to the complexity of the interactions between host plants and viruses, which involve multiple physiological processes, transcriptomic analysis has become a crucial step in studying the mechanisms of these interactions. RNA sequencing (RNA-Seq) provides a powerful tool for identifying differentially expressed genes (DEGs) [5,17]. The transcriptomic changes induced by Obuda pepper virus (ObPV, a tobamovirus), PMMoV, pepper chlorosis virus (CaCV, a tospovirus), and CMV in pepper plants have been comprehensively analyzed [5,17,18,19,20]. However, the transcriptomic differences caused by ToBRFV infection in pepper have not yet been studied. In this research, we used RNA-Seq technology to analyze changes in gene expression between healthy leaves and ToBRFV-infected leaves. Our results provide transcriptome-wide insights into the molecular basis of pepper leaf resistance to ToBRFV infection and offer potential candidate genes for improving resistant varieties.

## 2. Results

### 2.1. Preparation of Transcriptome Samples from ToBRFV-Infected Pepper Leaves

**Figure 1:**
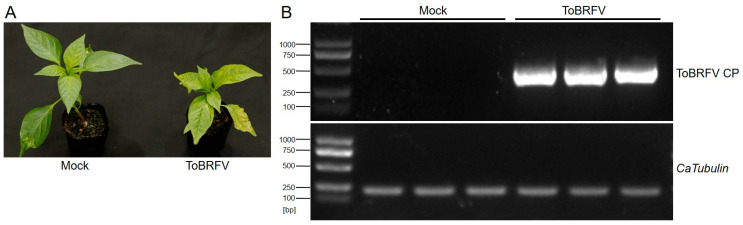
Symptoms of ToBRFV infection in pepper 13 days post-inoculation and viral RNA detection in the top leaves. (**A**) Symptoms in ‘Haonong 11’ pepper 13 days after ToBRFV inoculation. (**B**) Detection of viral capsid RNA in the top leaves of pepper from both mock and virus-inoculated groups, with *CaTubulin* as the reference gene.

Before germination, the seeds of the pepper variety “Haonong 11” were tested for ToBRFV infection using RT-PCR (ToBRFV CP primer sequences are listed in Table S1). Seeds confirmed to be free of ToBRFV were used for germination and subsequent transcriptome sequencing experiments. After 25 days of germination (with the plants kept in enclosed insect-proof nets), the experimental group was mechanically inoculated by rubbing the first pair of true leaves with ToBRFV-infected sap, while the control group was treated with the same method using phosphate-buffered saline (PBS) buffer. Thirteen days post-inoculation, the ToBRFV-inoculated plants exhibited symptoms such as leaf curling, chlorosis, and growth stagnation in the apical leaves, whereas the control plants developed normally (Figure 1A). Prior to formal sampling, a small number of newly emerged apical leaves from both the experimental and control groups were collected for RT-PCR analysis using ToBRFV CP primers (Table S1). The results confirmed that the experimental group was successfully infected with ToBRFV, while the control group remained uncontaminated (Figure 1B). Subsequently, three biological replicates from both the inoculated and control groups were collected and sent to Beijing Novogene Bioinformatics Technology Co., Ltd. (Beijing, China) for high-throughput RNA-Seq.

### 2.2. Sequencing and De Novo Assembly of Transcriptome

In this study, RNA-Seq was performed using the Illumina platform. The three control samples yielded 48,395,916, 40,271,370, and 47,622,208 raw reads, respectively, while the three virus-inoculated samples yielded 46,630,278, 44,595,956, and 44,872,350 raw reads, respectively (Table 1). After removing low-quality bases, short reads, and adapter sequences, the three control samples generated 47,391,258, 39,288,882, and 46,576,848 clean reads, respectively, and the three virus-inoculated samples generated 45,595,038, 43,575,074, and 43,617,228 clean reads, respectively, with a data efficiency rate exceeding 97% (Table 1). Using the HISAT2 (2.0.5) software, the overall read alignment rate to the pepper genome was over 88%, and the unique read alignment rate was above 84%. The Q20 and Q30 values were both greater than 92% (Table 1).

### 2.3. Identification of DEGs in Pepper Leaves Responding to ToBRFV

**Figure 2:**
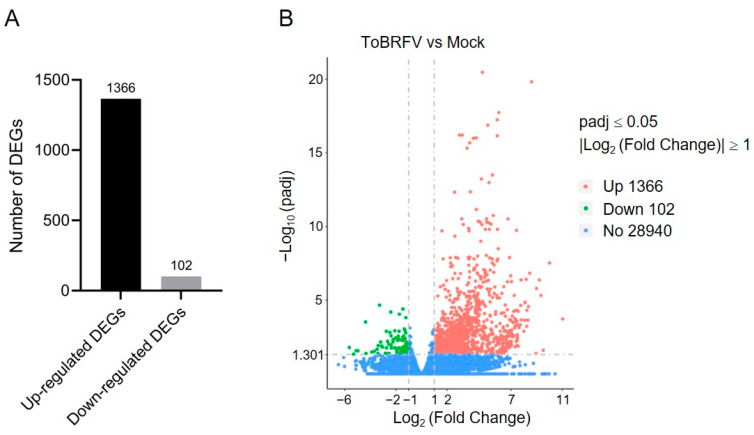
Statistical analysis of DEGs in pepper leaves after ToBRFV inoculation. (**A**) Bar chart showing the number of upregulated and downregulated genes in pepper leaves 13 days after ToBRFV inoculation. (**B**) Volcano plot displaying the number of DEGs in pepper leaves 13 days after ToBRFV inoculation.

By comparing the sequencing data of the ToBRFV-infected group with the control group using DESeq2, we identified DEGs in response to ToBRFV infection. The criteria for screening DEGs were |log_2_ (Fold Change)| ≥ 1 and padj ≤ 0.05. A total of 1468 DEGs were identified in the ToBRFV-infected group, of which 1366 were upregulated (accounting for 93%) and 102 were downregulated (accounting for 7%) (Figure 2A,B; Table S2). Furthermore, the expression of 28,940 genes showed no significant difference between the control group and the virus-infected group (Figure 2B).

### 2.4. GO and KEGG Enrichment Analysis of DEGs

**Figure 3:**
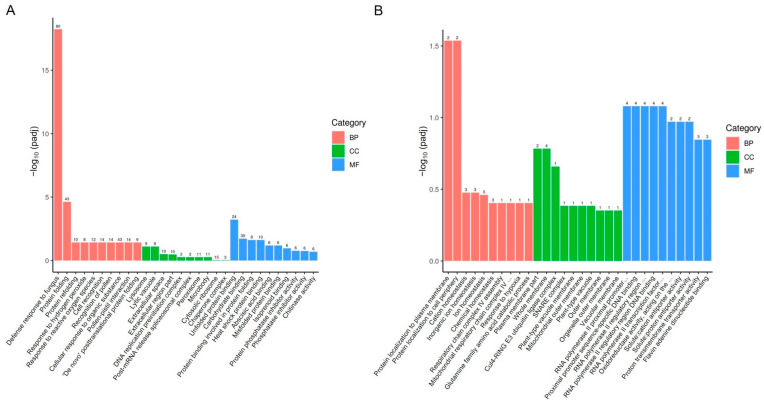
GO enrichment analysis of DEGs in pepper leaves 13 days after ToBRFV inoculation. (**A**) GO enrichment analysis of upregulated genes. (**B**) GO enrichment analysis of downregulated genes. The horizontal axis represents GO Terms, while the vertical axis indicates the significance level of GO Term enrichment, expressed as −log_10_ (padj). The values on the columns represent the number of DEGs enriched for that term, with different colors representing the three GO subcategories: BP, CC, and MF.

To analyze the functions of the DEGs, we performed Gene Ontology (GO) analysis on both upregulated and downregulated DEGs. GO is a comprehensive database that describes gene functions and is divided into three categories: biological process (BP), cellular component (CC), and molecular function (MF). For significantly upregulated genes, the enriched biological processes included defense response to fungus, protein folding, response to hydrogen peroxide, and response to reactive oxygen species (Figure 3A and Figure S1). The associated cellular components included lysosome, lytic vacuole, extracellular space, and cytosolic ribosome (Figure 3A). The molecular functions included unfolded protein binding, carbohydrate binding, and heat shock protein binding (Figure 3A). For significantly downregulated genes, the enriched biological processes included protein localization to the plasma membrane, protein localization to the cell periphery, cation homeostasis, and response to hypoxia (Figure 3B and Figure S1). The associated cellular components included plasma membrane parts, whole membranes, and Cul4-RING E3 ubiquitin ligase complexes (Figure 3B). The molecular functions included RNA polymerase II proximal promoter sequence-specific DNA binding, oxidoreductase activity, solute:cation antiporter activity, and proton transmembrane transporter activity (Figure 3B).

**Figure 4:**
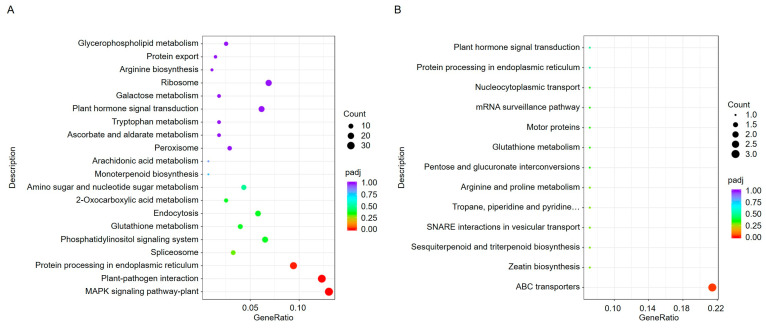
KEGG enrichment analysis of DEGs in pepper leaves 13 days after ToBRFV inoculation. (**A**) KEGG enrichment analysis of upregulated genes. (**B**) KEGG enrichment analysis of downregulated genes. The horizontal axis represents the ratio of the number of DEGs annotated to a KEGG pathway to the total number of DEGs. The vertical axis displays the KEGG pathways. The dot size corresponds to the number of genes annotated to each KEGG pathway, while the color gradient (from red to purple) indicates the significance level of the enrichment.

To further understand the biological processes in which these DEGs are involved, we used the Kyoto Encyclopedia of Genes and Genomes (KEGG) database to analyze pathway annotations. KEGG is an integrated database that combines genomic, chemical, and systemic functional information. Through pathway enrichment analysis, a total of 76 pathways were identified. The upregulated genes in ToBRFV-infected plants were primarily associated with pathways such as the MAPK signaling pathway, plant-pathogen interaction, protein processing in the endoplasmic reticulum (ER), and spliceosome (Figure 4A). The downregulated genes were mainly involved in pathways such as ABC transporters (Figure 4B).

### 2.5. Validation of DEGs by Quantitative PCR

**Figure 5:**
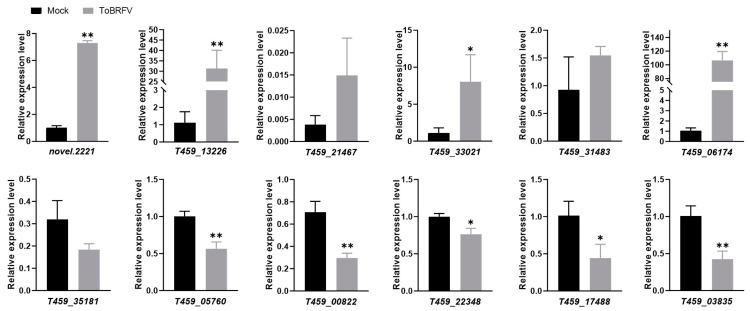
Validation of selected gene expression from RNA-Seq data using real-time quantitative PCR. Statistical analysis was performed using a Student’s *t*-test, with bar graphs representing mean ± SD. The asterisk indicates a statistically significant difference between the ToBRFV-inoculated group and the control group (* *p* < 0.05, ** *p* < 0.01). The experimental results were consistent across three repeated experiments.

To validate the transcriptome sequencing data, we selected six upregulated and six downregulated DEGs for RT-qPCR validation. These genes are involved in various functions, including disease resistance, transcription factor activity, and plant metabolism (Table 2). The quantitative PCR results showed that among the selected upregulated genes, *novel.2221*, *T459_13226*, *T459_33021*, and *T459_06174* were significantly upregulated, consistent with the trends observed in the transcriptome data (Figure 5; Table S3). However, *T459_21467* and *T459_31483* were only slightly upregulated, with smaller changes compared to the transcriptome data (Figure 5; Table S3). Among the downregulated genes, *T459_05760*, *T459_00822*, *T459_22348*, *T459_17488*, and *T459_03835* were significantly downregulated, aligning with the transcriptome data trends (Figure 5; Table S3). In contrast, *T459_35181* was only slightly downregulated, with a smaller change than indicated by the transcriptome data (Figure 5; Table S3).

Overall, the RT-qPCR results were consistent with the high-throughput sequencing data, demonstrating that RNA-Seq is a reliable method for identifying and selecting DEGs induced by ToBRFV infection in pepper plants.

## 3. Discussion

Since its initial discovery, ToBRFV has rapidly spread worldwide, causing severe impacts on the global pepper and tomato industries [10,11]. As a result, ToBRFV is recognized as a global plant quarantine disease [11]. During viral infection, rapid and comprehensive transcriptional reprogramming in host plant cells is crucial for defending against viral attacks. Understanding the mechanisms by which plants resist viruses and designing virus-resistant crops based on this knowledge is the safest and most environmentally friendly approach. However, our understanding of the molecular processes underlying plant responses to viral infections remains limited. Transcriptomic and expression profiling data provide valuable resources for gaining deeper insights into how host plants respond to viral infections. Currently, transcriptomic analyses of pepper plants infected with PMMoV, CMV, ObpV, and CaCV have been conducted [5,17,18,19,20], but the transcriptomic response of pepper plants to ToBRFV infection has not yet been reported. In this study, by analyzing pepper samples 13 days after ToBRFV infection, we identified 1468 DEGs, including 1366 upregulated and 102 downregulated genes. These findings provide a data foundation for further identifying host genes that play important roles in the ToBRFV-host interaction process.

Following ToBRFV infection in pepper, the top five most significantly enriched biological processes among upregulated genes were defense response to fungus, protein folding, protein refolding, response to hydrogen peroxide, and response to reactive oxygen species (Figure 3A and Figure S1). The top five enriched processes among downregulated genes were protein localization to the plasma membrane, protein localization to the cell periphery, cation homeostasis, inorganic ion homeostasis, and ion homeostasis (Figure 3B and Figure S1). In tomato (Moneymaker), ToBRFV infection at 21 days post-inoculation induced 270 upregulated genes and 252 downregulated genes [21]. The top five enriched processes among upregulated genes were multicellular organismal process, nucleobase-containing compound transport, cell recognition, pollination, and pollen-pistil interaction [21]. Among downregulated genes, the top five enriched processes were response to wounding, response to stress, lipid biosynthetic process, protein folding, and defense response [21]. These results suggest that pepper may be more sensitive to ToBRFV, triggering a stronger immune response. The relatively few downregulated genes in pepper, which were primarily related to ion homeostasis (e.g., cation homeostasis), may indicate that the virus disrupts ionic equilibrium to facilitate replication. The enrichment of protein folding/refolding processes suggests that ToBRFV infection induces ER stress, prompting the host to repair misfolded proteins. In contrast, tomato exhibited a more balanced gene expression response, possibly indicating greater tolerance to ToBRFV. The downregulation of genes involved in stress responses (e.g., wounding, defense) implies that the virus may actively suppress tomato’s defense pathways. The inhibition of lipid biosynthesis may reflect viral suppression of host defenses (e.g., jasmonate signaling) to promote infection. Notably, the downregulation of protein folding in tomato—opposite to the trend in pepper—suggests that tomato does not undergo severe ER stress upon infection. Upregulated genes in tomato were enriched in multicellular processes and pollination-related pathways (e.g., pollen-pistil interaction), indicating activation of developmental pathways, possibly as a compensatory mechanism to mitigate viral damage. Although tomato and pepper both belong to the *Solanaceae* family, their responses to ToBRFV differ significantly. This divergence may stem from distinct immune strategies (pepper favoring an aggressive defense response, while tomato prioritizes growth-defense balance) and host-specific manipulation by viral-encoded proteins. These differences provide potential targets for designing virus-resistant crops and guiding breeding strategies.

Zhang et al. compared the transcriptomic data of PMMoV-infected tolerant and susceptible pepper varieties and found that the mitogen-activated protein kinase (MAPK) signaling pathway, plant-pathogen interaction pathway, and flavonoid biosynthesis pathway were commonly activated in both varieties. Additionally, through weighted gene co-expression network analysis (WGCNA), seven hub genes were identified [5]. In this study, KEGG enrichment analysis of DEGs in ToBRFV-infected pepper plants revealed significant activation of the MAPK pathway and plant-pathogen interaction pathway (Figure 4), consistent with the transcriptomic enrichment results observed in ToBRFV-infected tomato plants [21]. In eukaryotes, the MAPK cascade is a highly conserved signaling module typically composed of a MAPK, a MAPK kinase (MAPKK), and a MAPK kinase kinase (MAPKKK), positioned downstream of receptors/sensors that convert external stimuli into intracellular responses. In plants, the MAPK cascade plays a critical role in signaling defense against pathogen attacks. The activation of MAPKs is one of the earliest signaling events following the perception of pathogen-associated molecular patterns (PAMPs) and pathogen effectors [22,23]. Our findings suggest that the MAPK pathway is also involved in signaling plant defense against viral infections.

Zhu et al. utilized Illumina and single-molecule real-time (SMRT) RNA-Seq technologies to analyze transcriptomic data from hot pepper at five different time points after CMV infection, identifying a total of 2143 DEGs. GO and KEGG analyses revealed that these genes were involved in pathways related to plant stress responses, defense responses, and plant-pathogen interactions. Additionally, the expression patterns of some leucine-rich repeat receptor-like serine/threonine protein kinases (LRR-RLKs) were altered during CMV infection [20]. In the transcriptomic data induced by ToBRFV infection in pepper, we also observed significant changes in the expression levels of numerous RLK genes, with 143 upregulated and 3 downregulated (Table S2). The kinase types included LRR-RLK, G-type lectin S-receptor-like serine/threonine-protein kinase, cysteine-rich receptor-like protein kinase, proline-rich receptor-like protein kinase, and L-type lectin-domain-containing receptor kinase, among others (Table S2). This suggests that RLKs also play an important role in plant responses and defense mechanisms against viral infections. The specific functions of these RLKs and the signaling pathways they regulate warrant further in-depth investigation.

Jiao et al. analyzed the transcriptomic data of pepper plants infected with PMMoV and found that many autophagy-related genes (ATGs) were slightly upregulated. Confocal microscopy observations revealed the formation of double-membrane autophagic structures in PMMoV-infected plants. Additionally, autophagy inhibitors significantly increased the accumulation of viral RNA in plants, indicating that autophagy regulates pepper plant resistance to PMMoV [17]. In our transcriptomic data of pepper plants, we identified 27 autophagy-related proteins, but their expression levels did not show significant changes after ToBRFV infection. Current research suggests that autophagy protects plants from infections by viruses such as cotton leaf curl Multan virus (CLCuMuV), tomato yellow leaf curl virus (TYLCV), tomato yellow leaf curl China virus (TYLCCNV), barley stripe mosaic virus (BSMV), and turnip mosaic virus (TuMV) [24]. However, the extent to which the autophagy pathway is involved in plant defense against ToBRFV infection remains unclear and warrants further investigation, making it a promising direction for future research.

Alternative splicing (AS) plays a role in most plant processes, such as growth, development, and responses to external signals. The AS of initial host transcripts allows a single eukaryotic gene to produce multiple transcripts, thereby enhancing transcript and protein diversity [25,26]. Current research has identified changes in AS of host transcripts in plants infected with several viruses and viroids [26,27,28,29,30]. This study also found that some pepper genes underwent AS events following ToBRFV infection, with the types of AS involved including Skipped Exon (SE) and Mutually Exclusive Exon (MXE). The AS genes identified in this study following ToBRFV infection in pepper plants lay a solid foundation for future research into the importance of host transcript AS in determining the outcomes of virus-host interactions.

## 4. Materials and Methods

### 4.1. Plant Cultivation and ToBRFV Inoculation

Pepper plants (variety: Haonong 11) were cultivated in a greenhouse under a 16 h light/8 h dark cycle at 24 °C. The plants were entirely covered with insect-proof nets to prevent pathogen infections and insect bites. Approximately 25 days after pepper seed germination, the plants were mechanically inoculated by rubbing the leaves with either ToBRFV viral particle-containing plant sap or PBS as a control. The ToBRFV strain was isolated from Yuanmou County, Yunnan Province, China. At 13 days post-inoculation (dpi), small tissue samples were collected from the apical leaves of both ToBRFV-infected and mock-inoculated control plants. Total RNA was extracted using TRIzol reagent and reverse transcribed into cDNA. Viral infection status was confirmed by RT-PCR with ToBRFV CP-specific primers, verifying successful infection in the experimental group while confirming the absence of viral contamination in controls. Subsequently, newly emerged apical leaf samples from both groups were collected for high-throughput sequencing, with three biological replicates per group.

### 4.2. Transcriptome Sequencing Library Construction, Quality Control, and Sequencing

The initial RNA for library construction was total RNA extracted from the samples (TIANGEN, Cat. No. DP441, Beijing, China). Poly(A) mRNA was captured using the mRNA Capture Module (ABclonal, Cat. No. RK20340, Wuhan, China) and fragmented. Subsequently, the Fast RNA-seq Lib Prep Kit V2 (ABclonal, Cat. No. RK20306, Wuhan, China) was used for first-strand cDNA synthesis, second-strand cDNA synthesis, adapter ligation, purification of the ligation products, library amplification, and purification of the PCR products, ultimately resulting in the acquisition of the library. Subsequently, preliminary quantification was performed using a Qubit 2.0 Fluorometer (Thermo Fisher Scientific, Waltham, MA, USA), and the library was diluted to 1.5 ng/µL. The insert size of the library was then detected using an Agilent 2100 bioanalyzer (Agilent Technologies, Santa Clara, CA, USA). Once the insert size met expectations (250–300 bp), the effective concentration of the library was accurately quantified using RT-qPCR (the effective concentration needed to be above 1.5 nM) to ensure library quality. After passing quality control, libraries were pooled according to their effective concentrations and the desired sequencing data volume, followed by Illumina sequencing (NovaSeq X Plus platform, San Diego, CA, USA).

### 4.3. Data Quality Control

Sequencing reads generated by the high-throughput sequencer (150 bp read length) were converted from image data into sequence data (reads) using CASAVA base calling and stored in FASTQ format. These files primarily contain sequence information and corresponding quality scores. To ensure the quality and reliability of data analysis, raw data were filtered using fastp (version 0.23.1) to remove reads containing adapters, reads with ambiguous bases (N), and low-quality reads (reads with more than 50% of bases having a Qphred score ≤ 5). This process yielded clean data. Additionally, the quality of the clean data were assessed by calculating Q20, Q30, GC content, and sequence duplication levels. All downstream analyses were performed using the high-quality clean data.

### 4.4. Alignment of Sequences to the Reference Genome

The pepper reference genome and gene model annotation files were downloaded from the EnsemblPlants genome database (https://www.ebi.ac.uk/ena/browser/view/GCA_000512255.2, accessed on 21 April 2025). The high-quality clean reads were then aligned to the reference genome using HISAT2 software (version 2.0.5) with default parameters to obtain precise mapping information of the reads on the reference genome.

### 4.5. Gene Expression Quantification and Differential Expression Analysis

Based on the alignment information of reads to the reference genome, FeatureCounts was used to count the number of reads covering each gene (including newly predicted genes) from the start to the end positions. Reads with alignment quality scores below 10, non-paired reads, and reads mapped to multiple regions of the genome were filtered out. The FPKM (Fragments Per Kilobase of transcript per Million mapped reads) value for each gene was then calculated based on gene length. Differential expression analysis between the experimental and control groups was performed using the DESeq2 software (version 1.20.0). The Benjamini and Hochberg method was applied to adjust the *p* values to control the false discovery rate. Genes that were determined by |log_2_ (Fold Change)| ≥ 1 with adjusted *p* values ≤ 0.05, as identified by DESeq2, were assigned as DEGs.

### 4.6. DEGs GO and KEGG Enrichment Analysis

The clusterProfiler software (version 3.8.1) was used to perform GO functional enrichment analysis and KEGG pathway enrichment analysis on the DEG sets. GO is a comprehensive database describing gene functions, divided into three categories: biological process, cellular component, and molecular function. GO functional enrichment was considered significant with a padj value less than 0.05. KEGG is an integrated database combining genomic, chemical, and systemic functional information. KEGG pathway enrichment was considered significant with a padj value less than 0.05.

### 4.7. Real-Time Quantitative PCR (RT-qPCR)

Total RNA was extracted from ToBRFV-infected (or mock-inoculated) leaves using TRIzol reagent (Thermo Fisher Scientific, Carlsbad, CA, USA). Approximately 1 µg of total RNA was reverse-transcribed into cDNA using the TransScript One-Step gDNA Removal and cDNA Synthesis SuperMix kit from TransGen Biotech (Cat. No. AT311-03, Beijing, China). Real-time quantitative PCR was then performed on the cDNA using the Applied Biosystems QuantStudio™ 5 System (Thermo Fisher Scientific, Foster City, CA, USA). Twelve DEGs were selected from RNA-seq results, and gene-specific primers were designed to analyze their expression changes. The relative gene expression changes were calculated using the 2^−∆∆CT^ method [31], with the *CaTubulin* gene serving as the internal reference. The primer sequences used in RT-qPCR are shown in Table S1.

## 5. Conclusions

This study analyzed the gene expression profile of pepper leaves 13 days after infection with ToBRFV and mock inoculation using RNA-Seq technology for the first time and identified 1468 DEGs. Functional analysis indicated that the DEGs were involved in biological processes such as plant defense responses, protein folding, and plant-pathogen interactions. RT-qPCR validation of several DEGs revealed that the trend of changes was consistent with the RNA-Seq data. Our systematic analysis provides a molecular basis for the pepper leaf response to ToBRFV infection at the transcriptomic level and offers valuable new insights for research into the pathogenesis of ToBRFV and the identification of genes related to pepper resistance to ToBRFV infection.

## Figures and Tables

**Table 1:** Statistics description of RNA-Seq data 13 days post-ToBRFV inoculation in pepper leaves.

Sample	Mock-1	Mock-2	Mock-3	ToBRFV-1	ToBRFV-2	ToBRFV-3
Raw_Read	48,395,916	40,271,370	47,622,208	46,630,278	44,595,956	44,872,350
Clean_reads	47,391,258	39,288,882	46,576,848	45,595,038	43,575,074	43,617,228
Effective (%)	97.92	97.56	97.80	97.78	97.71	97.20
Total_Map (%)	42,306,124 (89.27)	34,720,751 (88.37)	41,700,214 (89.53)	40,875,579 (89.65)	38,642,689 (88.68)	38,950,973 (89.30)
Unique_Map (%)	40,382,064 (85.21)	33,304,170 (84.77)	39,960,963 (85.80)	39,548,312 (86.74)	36,923,883 (84.74)	37,406,207 (85.76)
Q20 (%)	98.3	97.26	97.45	97.65	97.34	97.53
Q30 (%)	95.34	92.88	93.25	93.71	93.04	93.35
GC Contents (%)	42.92	42.58	42.85	42.88	43.11	43.14

**Table 2:** List of 12 candidate genes validated by quantitative PCR.

Gene Name	Description	CK_1	CK_2	CK_3	13d_1	13d_2	13d_3	log_2_ (Fold Change)	Regulation
*novel.2221*	Receptor kinase-like protein	0.0	0.0	0.0	71.0	14.3	170.5	9.0	Up
*T459_13226*	Defensin-like protein	0.0	0.0	0.0	1.3	75.6	14.4	7.5	Up
*T459_21467*	Putative WRKY transcription factor	7.9	14.3	6.7	4333.1	242.0	386.0	7.4	Up
*T459_33021*	Ethylene-responsive transcription factor	0.0	0.0	0.0	32.9	4.1	1.0	6.2	Up
*T459_31483*	Very-long-chain aldehyde decarbonylase	0.0	0.0	0.0	78.9	4.1	7.7	7.5	Up
*T459_06174*	Acidic endochitinase	17.6	50.2	9.2	5989.3	579.9	1756.8	6.8	Up
*T459_35181*	Putative disease resistance protein	25.6	28.7	24.4	3.9	0.0	4.8	−3.2	Down
*T459_05760*	Calcium-binding protein	181.5	149.5	126.9	56.6	66.4	90.0	−1.1	Down
*T459_00822*	Transcription factor MYB111	330.4	222.2	239.5	18.4	17.4	96.8	−2.6	Down
*T459_22348*	Transcription factor bHLH96	419.4	436.3	439.5	101.3	190.9	194.5	−1.4	Down
*T459_17488*	Probable 2-oxoglutarate-dependent dioxygenase	27.3	19.5	26.0	5.3	0.0	3.8	−3.0	Down
*T459_03835*	Long-chain-alcohol oxidase	133.0	114.7	95.0	10.5	30.6	23.0	−2.4	Down

The table presents the differential expression analysis results of the selected 12 genes. CK_1 to CK_3 and 13d_1 to 13d_3 represent the normalized read count values for each sample. The log_2_ (Fold Change) indicates the ratio of gene expression levels between the virus-infected group and the control group, which was processed by the shrinkage model of the differential analysis software and then log_2_-transformed.

## Data Availability

Sequencing data from this article were deposited at the CNGB Sequence Archive (CNSA) of China National GeneBank Database (CNGBdb) with accession number CNP0006881.

## Supplementary Materials

The following supporting information can be downloaded at: https://www.mdpi.com/article/10.3390/plants14091280/s1, Figure S1: Directed acyclic graph (DAG) showing the results of GO enrichment analysis for BP. (A) DAG shows the GO enrichment analysis results (BP) of upregulated DEGs. B. DAG shows the GO enrichment analysis results (BP) of downregulated DEGs. Each node in the DAG represents a GO term (BP). The box-shaped nodes highlight the top 5 most significantly enriched GO terms, with color intensity indicating the enrichment strength—darker shades denote higher enrichment significance. Each node is labeled with the GO term name and its adjusted *p*-value (padj) from the enrichment analysis. Table S1: Primer sequences used in this study; Table S2: List of DEGs induced 13 days after ToBRFV infection in Haonong 11; Table S3: The original data corresponding to the RT-qPCR results in Figure 5.
