# Supplementary figures and images for: Transcriptome Analysis of Pepper Leaves in Response to Tomato Brown Rugose Fruit Virus Infection
Source: Plants (Basel). 2025 Apr 23;14(9):1280. doi: 10.3390/plants14091280 (PMC12073185; doi:10.3390/plants14091280)

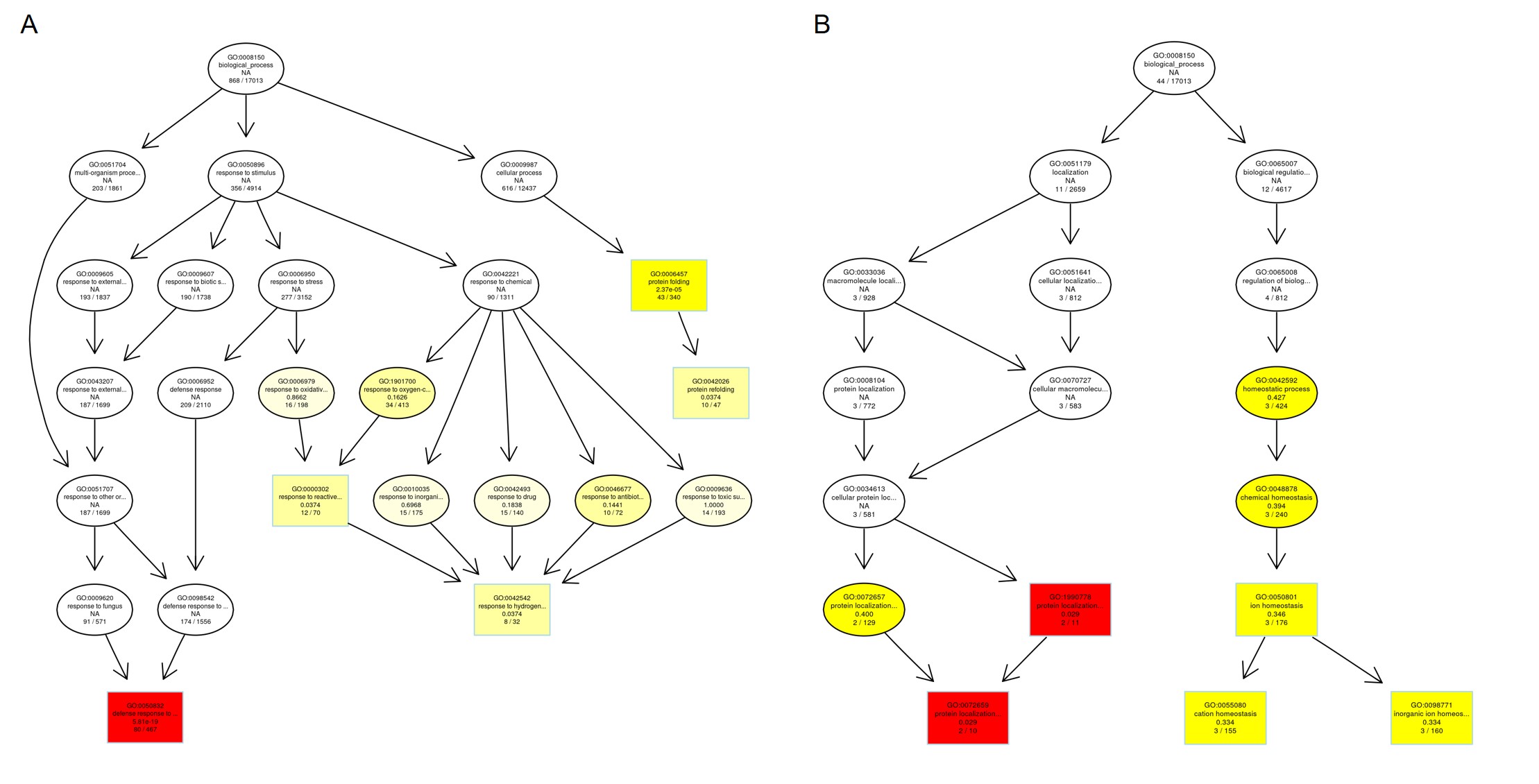

Supplement: Supplementary file 1 [file plants-14-01280-s001.zip › Figure S1.jpg]
